# Exploring lipid–protein interactions in plant membranes

**DOI:** 10.1093/jxb/erae199

**Published:** 2024-05-06

**Authors:** Eliška Škrabálková, Přemysl Pejchar, Martin Potocký

**Affiliations:** Institute of Experimental Botany of the Czech Academy of Sciences, Prague, Czech Republic; Department of Experimental Plant Biology, Faculty of Science, Charles University, Prague, Czech Republic; Institute of Experimental Botany of the Czech Academy of Sciences, Prague, Czech Republic; Institute of Experimental Botany of the Czech Academy of Sciences, Prague, Czech Republic; University of South Bohemia in České Budějovice, Czech Republic

**Keywords:** Genetically encoded biosensors, lipid manipulation, membrane lipid imaging, microscopy, peripheral membrane proteins, protein–lipid interactions

## Abstract

Once regarded as mere membrane building blocks, lipids are now recognized as diverse and intricate players that mold the functions, identities, and responses of cellular membranes. Although the interactions of lipids with integral and peripheral membrane proteins are crucial for their localization, activity, and function, how proteins bind lipids is still far from being thoroughly explored. Describing and characterizing these dynamic protein–lipid interactions is thus essential to understanding the membrane-associated processes. Here we review the current range of experimental techniques employed to study plant protein–lipid interactions, integrating various methods. We summarize the principles, advantages, and limitations of classical *in vitro* biochemical approaches, including protein–lipid overlays and various liposome binding assays, and complement them with *in vivo* microscopic techniques centered around the use of genetically encoded lipid sensors and pharmacological or genetic membrane lipid manipulation tools. We also highlight several emerging techniques still awaiting their advancement into plant membrane research and emphasize the need to use complementary experimental strategies as key for elucidating the mechanistic roles of protein–lipid interactions in plant cell biology.

## Teaser

The binding of proteins to membrane lipids is vital for membrane biological functions. We review the multifaceted methodological approaches aimed at identifying, understanding, and validating protein–lipid interactions in plant cells.

## Introduction

Proteins and lipids, the essential components of cellular membranes, play vital roles in structuring all living cells. Lipids, the fundamental building blocks of cell membranes, are abundant and highly diverse in their structures and functions. Lipids in all eukaryotic membranes, including plants, consist of three main classes: glycerolipids, sphingolipids, and sterols. The most abundant are glycerolipids, which are divided into four groups: phospholipids, galactolipids, triacylglycerols, and sulfolipids (Reszczyńska and Hanaka, 2020). Phospholipids, amphipathic assemblies of hydrophobic fatty acids and hydrophilic headgroups linked via a glycerol backbone and phosphate group, possess several features that give them characteristic geometry and size. The simplest phospholipid class, with only a phosphate for a headgroup, is phosphatidic acid (PA). This simple phosphate headgroup can be further modified to form the different classes of phospholipids by the addition of a choline, serine, ethanolamine, glycerol, or inositol molecule, resulting in phosphatidylcholine (PC), phosphatidylserine (PS), phosphatidylethanolamine (PE), phosphatidylglycerol (PG), or phosphatidylinositol (PI) lipids, respectively (Nakamura, 2017; Colin and Jaillais, 2020).

Lipids can interact with proteins, either covalently or non-covalently, to regulate further lipid metabolism, transport, and signaling, as well as to modulate the cellular activities and locations of proteins. Crucially, specific protein–lipid interactions are essential for the integration and biological activity of both integral and peripheral proteins in the membranes (Sekereš *et al.*, 2015). Hence, similar to protein–protein and protein−nucleic acid interactions, deciphering the lipid–protein interactomes is necessary to better understand the roles lipids play in various cell mechanisms. Unlike proteins, lipids are not directly encoded by genes and, given the non-covalent nature of the lipid bilayers, their dynamic structures are diverse and complex. Notably, alongside many proteins in a cellular system, there are multitudes of functionally relevant lipid–protein interacting events. As a result, the systematic characterization of lipid–protein interactions is more challenging when compared with other types of interactions (Ge *et al.*, 2022).

**Fig. 1.**
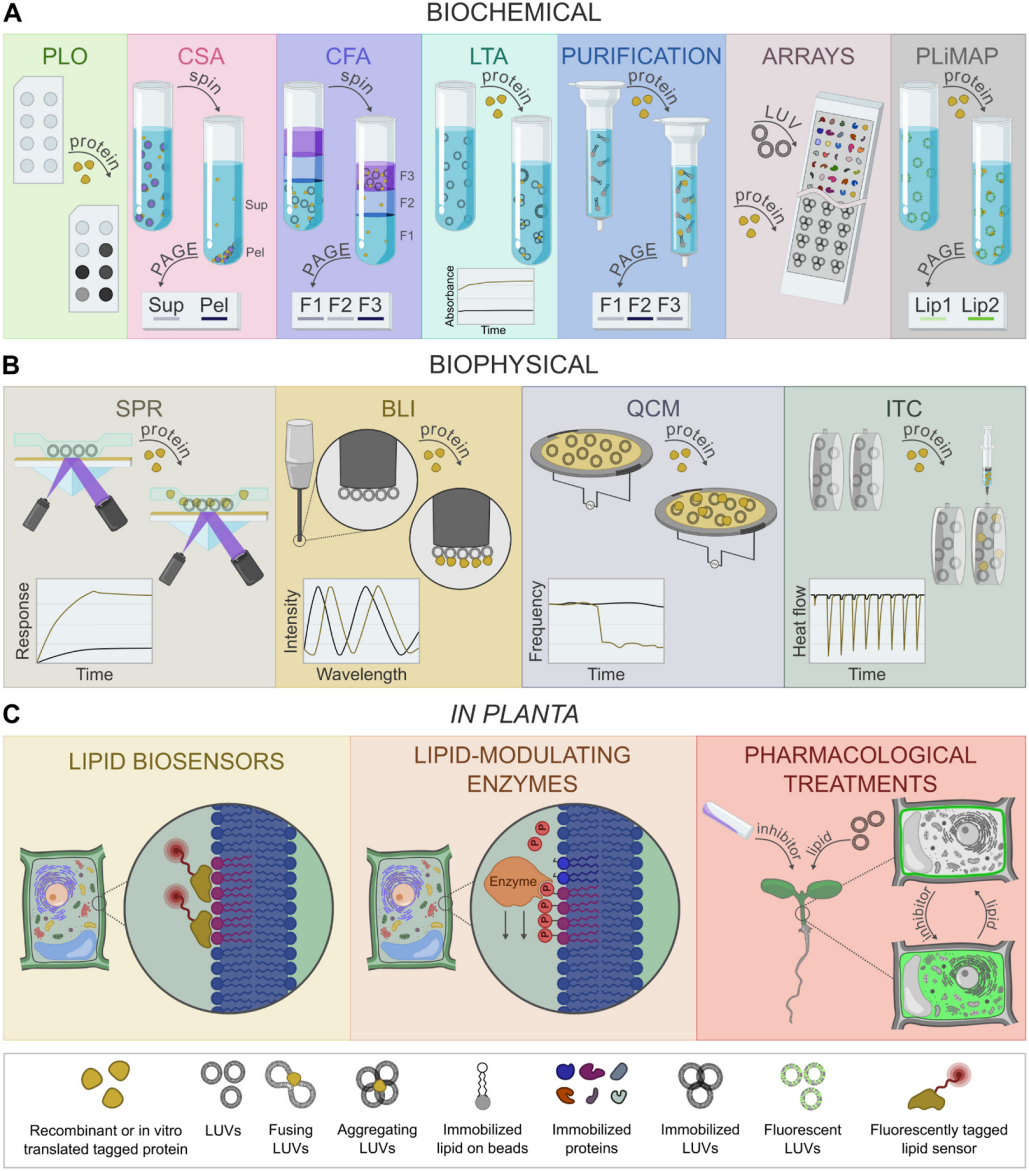
Schematic representation of selected methods used to analyze protein–lipid interaction. (A) Variety of predominantly liposome-based biochemical methods unraveling the lipid binding potential of the examined protein and ways of detection. PLiMAP and microarrays are recently established methods with possible implementation in plant cell biology. (B) Selected biophysical methods that quantitatively analyze lipid–protein interactions. Small insets depict primary outcomes of analyses. (C) Examples of *in planta* methods represented by genetically encoded sensors, lipid-modifying enzymes, or external treatments. BLI, bio-layer interferometry; CFA, co-flotation assay; CSA, co-sedimentation assay; F, fraction; ITC, isothermal titration calorimetry; LTA, liposome turbidity assay; LUV, large unilamellar vesicles; Pel, pellet; PLiMAP, proximity-based labeling of membrane-associated proteins; PLO, protein–lipid overlay; QCM, quartz-crystal microbalance; SPR, surface plasmon resonance; Sup, supernatant.

Here, we provide a brief introduction to currently available experimental methods for detecting, quantifying, and validating protein–lipid interactions that are established in plants and present several experimental approaches or tools whose introduction would be valuable to plant membrane research. While we recognize the importance and power of computational methods to reveal mechanistic details of lipid–protein interactions, for the sake of space, we refer the reader to the recent review covering this topic (Neubergerová and Pleskot, 2024). We structure the review according to the nature of the methods used to analyze the protein–lipid interactions, categorizing them into biochemical, biophysical, and microscopic approaches (Fig. 1), discussing the basic principles and applications, and highlighting their advantages or limitations (Table 1). Since this is a method-oriented review, we only refer to selected examples of lipid-binding proteins, primarily to illustrate the diversity of techniques studying protein–lipid interactions (Table 1). For more detailed in-depth coverage of plant proteins binding individual lipids, we refer the readers to specialized reviews (Stace and Ktistakis, 2006; McLoughlin *et al.*, 2013; Kim and Wang, 2020; de Jong and Munnik, 2021; Marković and Jaillais, 2022; Goldy and Caillaud, 2023).

**Table 1.** Tools for the identification, quantification, and validation of protein–lipid interactions, including examples of determined lipid binding to selected plant proteins

Technique	Principle	Advantages	Limitations	Lipid-binding proteins	Lipids bound	References^*a*^
Protein lipid overlay (PLO) assay^*b*^	WB-based method used with recombinant or *in vitro* produced proteins; detected by ECL	Defined and easy to use protocols; rapid outcomes	Lipids in non-physiological state; only qualitative results	MS1 SPHK1 ROP6 DTH1 ACBP2 LtpI-4	PA, PIPs PA PG PE lysoPC long FAs	Li *et al.* (2021) Pandit *et al.* (2018) Han *et al.* (2018) Lee *et al.* (2020) Gao *et al.* (2010) Deeken *et al.* (2016)
Co-sedimentation assay (CSA)^*b*^	Liposome-based method performed with recombinant or *in vitro* produced protein; binding proteins sediment; detected by SDS–PAGE	Defined and easy to use protocols; qualitative and quantitative results	Not usable for large proteins; sensitive towards lipid phase separation; low throughput	SCAB1 ATM2 OTU11 FtsZ1 NPC4 SPK1	PI3P PIPs PIPs PC, PG PC PS	Yang *et al.* (2022b) Kastner *et al.* (2022) Vogel *et al.* (2022) Liu *et al*. (2022) Fan *et al.* (2023) Mallery *et al.* (2022)
Co-flotation assay (CFA)^*b*^	Liposome-based method performed with recombinant proteins; binding proteins float to the top layer of the sucrose gradient; detected by SDS–PAGE	Qualitative and quantitative results; suitable for large proteins and complexes	Intricate and time-demanding preparation; low throughput	SYT1 ERD7 PDLP5 NAA60 DRP1A	PI(4,5)P_2_ PA SP PM-like liposomes	Qian *et al.* (2022) Barajas-Lopez *et al.* (2021) Liu *et al.* (2020) Linster *et al.* (2020) Backues and Bednarek (2010)
Liposome turbidity assay (LTA)^*b*^	Liposome-based method performed with recombinant proteins; detected by change in absorbance	Detection of membrane fusion events; suitable for integral membrane proteins	Indirect results with need for second method verification; specific instrumentation needed	DGD1 PLDRP1 BON1 LEA18 TGD2	PA PA PG, Ca^2+^ PC, PG PC	Kelly *et al.* (2016) Ufer *et al.* (2017) Wang *et al.* (2020) Hundertmark *et al.* (2011) Roston *et al.* (2011)
Proteome microarrays^*b*,*c*^	Proteome microarrays incubated with liposomes; detected by laser scanner records	Qualitative and quantitative results; high throughput	No plant-specific protocol; difficult array preparation and data analysis; specific instrumentation needed	NA	NA	Herianto *et al.* (2019, 2021) Saliba *et al.* (2016)
Liposome microarray-based assay (LiMA)^*b,c*^	Imobilized liposome arrays incubated with proteins; detected by automated microscope	Qualitative and quantitative results; high throughput	No plant-specific protocol; difficult array preparation and data analysis; specific instrumentation needed	NA	NA	Herianto *et al.* (2022) Saliba *et al.* (2014)
Proximity-based labeling of membrane-associated proteins (PLiMAP)^*b,c*^	Cross-linking of proteins with interacting liposomes; detected by SDS–PAGE	Qualitative results; suitable for high-molecular proteins and complexes	No plant-specific protocol; semi-quantitative; BDPE probe can affect the outcomes	NA	NA	Jose *et al.* (2020)
Affinity purification with immobilized lipids^*b*^	Lipid-coated beads loaded with purified proteins; lipid-binding proteins eluted and analyzed by WB or MS	Qualitative results; identification of quantity of lipid-binding proteins	Lipids in non-physiological state; complex preparation of immobilized lipids	TUA2 PPC1 ARGAH2 BTS	PA PA PA PA	Testerink *et al.* (2004) Testerink *et al.* (2004) Pandit *et al.* (2022) Pandit *et al.* (2022)
Surface plasmon resonance (SPR)^*b*^	Microfluidic system with proteins flowing across immobilized lipids; detecting molecular interactions via resonance change on plasmon surfaces	Qualitative and quantitative results; effective with low input protein concertation	Specific and expensive instrumentation needed; risk of clogging	LHY CCA1 SPHK1 SPHK2	PA PA PA PA	Kim *et al*. (2019) Kim *et al*. (2019) Guo *et al.* (2011) Guo *et al.* (2011)
Bio-layer interferometry (BLI)^*b*^	Immobilized liposomes at the tip of a sensor dipped into protein sample; measuring changes in the optical thickness and shift in interference upon protein lipid binding	High throughput; analysis of complex samples	Less sensitive; specific instrumentation needed	SYT1	PI(4,5)P_2_	Benavente *et al.* (2021)
Quartz-crystal microbalance (QCM)^*b*^	Lipids attached to the sensor chip surface probed with proteins; detected by changes in frequency of quartz resonator	Real-time binding kinetics; analysis of conformational changes, fibrillation, and hierarchical clustering	Need for two channel measurements to overcome unspecificity; specific instrumentation needed	SFH8	PI(4,5)P_2_	Liu *et al.* (2023)
Isothermal titration calorimetry (ITC)^*b*^	Two identical containers with high conductivity buffer containing lipids; proteins are titrated to one and both are measured for changes in conductivity upon binding event	Determination of binding constant, stoichiometry, entropy, and enthalpy; lipids in native state	Need for a high amount of pure lipids and proteins soluble in the same buffer	SPHK1 ABI1 GEF8 LEA18 ACBP2 LYSOPL2	PA PA PA PC, PG lysoPC lysoPC	Pandit *et al.* (2018) Mishra *et al.* (2006) Cao *et al.* (2017) Hundertmark *et al.* (2011) Miao *et al.* (2019) Miao *et al.* (2019)
Genetically encoded lipid sensors^*d*^	Lipid-specific biosensors; used for co-localization assays	Easy method; real-time localization of lipids	Competition with endogenous lipid-binding proteins; spatial–temporal limitations	ALA3 PLDδ3 DGK5 PSS1	PI4P, PS PA PA PS	Yang *et al.* (2022a) Pejchar *et al.* (2020) Kalachova *et al.* (2022) Yu *et al.* (2020)
Pharmacological treatment^*d*^	Lipid-specific drugs used for whole-plant treatment; visualization of protein relocalization	Easy method; Rapid real time response	Limited penetration to certain tissues; non-specific outcomes	MCTP TML PDK1 EXO70A1 NGR1	PI4P PI4P PI3P, PI4P PA, PI4P Sterols	Brault *et al.* (2019) Yperman *et al.* (2021) Tan *et al.* (2020) Synek *et al.* (2021) Kulich *et al.* (2024)
Lipid-derived affinity-based probes^*c*,*d*^	Lipid probe binds and labels interacting protein upon photoactivation; identification by MS	Proteome-wide identification of lipid-binding proteins	No plant-specific protocol; side effects from photoactivation; limited penetration into certain tissues	NA	NA	Ge *et al.* (2022) Shanbhag *et al.* (2023)
Photo-caged lipid probes^*c*^^,^^*d*^	Modified inactive lipid fused with photoremovable group is directed to certain tissue; upon photoactivation increases the levels of lipid at precise localization	Fast enrichment of lipid in distinct cellular membrane	No plant specific protocol; side effects from photoactivation and lipid overaccumulation; limited penetration into certain tissues	NA.	NA	Jiménez-López and Nadler (2023) Schultz (2023)
MAP-Sac1p^*d*^	Inducible system to deplete PI4P at the PM	Precise and fast manipulation at the PM; specificity for PI4P	Limitation to the inner leaflet of PM	ADR1 RPM1 NPH3 SYT1	PI4P PI4P PI4P PI4P	Saile *et al.* (2021) Saile *et al.* (2021) Reuter *et al.* (2021) Ruiz-Lopez *et al.* (2021)
Inducible depletion of PI(4,5)P_2_ in plants (iDePP)^*d*^	Inducible system to deplete PI(4,5)P_2_ at the PM	Precise and fast manipulation at PM; specificity for PI(4,5)P_2_	Limitation to the inner leaflet of PM	AP-2µ SH3P2	PI(4,5)P_2_ PI(4,5)P_2_	Doumane *et al.* (2021) Doumane *et al.* (2021)
*pss1-3* ^*d*^	Knockout mutant line without PS production	PS specific depletion	Severe phenotype; propagated as heterozygous	ROP6	PS	Platre *et al*. (2019)

ABI1, abscisic acid insensitive 1; ACBP2, acyl-CoA-binding protein 2; ADR1, activated disease resistance 1; ALA3, aminophospholipid ATPase 3; AP-2µ, adaptor protein complex 2 subunit µ; ARGAH2, arginine amidohydrolase 2; ATM2, Arabidopsis myosin 2; BON1, Bonzai 1; BTS, Brutus; CCA1, circadian clock associated 1; DGD1, diacylglycerol deficient 1; DGK5, diacylglycerol kinase 5; DRP1A, dynamin-related protein 1a; DTH1, delayed in TAG hydrolysis 1; ERD7, early-responsive to dehydration 7; EXO70A1, exocyst subunit Exo70 family protein A1; FAs, fatty acids; FtsZ1, filamenting temperature-sensitive mutant Z1; GEF8, guanine nucleotide exchange factor 8; LEA18, late embryogenesis abundant 18; LHY, late elongated hypocotyl; LtpI-4, lipid transfer protein 1-4; lysoPC, lysophosphatidylcholine; LYSOPL2, lysophospholipase 2; MCTP, multiple c2 domain and transmembrane region protein; MS1, male sterility 1; NA, not applicable; NAA60, *N*-acetyltransferase 60; NGR1, negative gravitropic response 1; NPC4, non-specific phospholipase C4; NPH3, non-phototropic hypocotyl 3; OTU11, ovarian tumor protease 11; PDK1, 3’-phosphoinositide-dependent protein kinase 1; PDLP5, plasmodesmata-located protein 5; PLDRP1, PLD regulated protein 1; PLDδ3, phospholipase Dδ3; PM, plasma membrane; PPC1, phosphoenolpyruvate carboxylase 1; PSS1, phosphatidylserine synthase 1; ROP6, Rho-related protein from plants 6; RPM1, resistance to *Pseudomonas syringae* pv *maculicola* 1; SCAB1, stomatal closure-related actin binding protein 1; SFH8, Sec fourteen-homolog 8; SH3P2, SH3 domain-containing protein 2; SP, sphingolipids; SPHK1, sphingosine kinase 2; SPHK2, sphingosine kinase 2; SPK1, Spike 1; SYT1, synaptotagmin 1; TGD2, trigalactosyldiacylglycerol 2; TML, TPLATE complex muniscin-like; TUA2, tubulin alpha 2: WB, western blotting.

^*a*^ Only selected reports containing the experiments performed in plants are listed. For methods not yet used in plants, representative reviews are cited.

^*b*^ *In vitro*-based technique.

^*c*^ Not yet tested in plants.

^*d*^ *In vivo*-based technique.

## Keep it (relatively) simple: biochemical approaches to uncovering protein– lipid interactions

Possibly the most significant advantage of biochemical methods for detecting protein–lipid interactions is their relative simplicity and the lack of the need for expensive equipment. On the other hand, they often provide only qualitative or semi-quantitative information, and their accuracy and sensitivity are often inferior compared with biophysical or microscopic techniques (Fig. 1A).

### Protein–lipid overlay

One of the simplest and most popular ways to examine protein–lipid interaction potential is by protein–lipid overlay (PLO) assay (Kubátová *et al.*, 2019; Han *et al.*, 2020; Kalachova *et al.*, 2022; Scholz *et al*., 2022). In a PLO assay, membranes spotted with lipids are firstly blocked with blocking proteins (e.g. BSA), incubated with an epitope-tagged target protein produced either in a recombinant system or by *in vitro* translation, and followed by a slightly modified western blotting-based assay (Han *et al.*, 2020). Crucial components are lipid-containing membrane strips that are either commercially available or can be prepared in the lab by spotting purified lipids onto polyvinylidene difluoride (PVDF) or nitrocellulose membranes. Despite the clear advantages, including the speed of analysis and a relatively large number of lipids that can be tested, PLO assay also has certain limitations that must be considered when interpreting the results. For instance, the lipids used in a PLO assay are dispersed on membranes in a non-physiological state, so the outcome is mostly qualitative data that do not allow determination of binding affinities, thermodynamics, or stoichiometry (Synek *et al.*, 2021; Scholz *et al.*, 2022). Also, commercial strips contain many non-plant lipids, which may further complicate the data interpretation. These limitations of the PLO assay render it mostly as an initial screening technique, which should be complemented with testing of a particular candidate lipid by additional methods with quantitative outcomes, such as liposome-based experiments.

### Liposome-based techniques

Liposomes, artificial vesicles containing one or more phospholipid bilayers, are the most widely employed and standard biomimetic systems used to study lipids, mimicking more physiological conditions (Herianto *et al.*, 2022). The possibility to create liposomes with variable lipid composition is another advantage (Julkowska *et al.*, 2013).

In a vesicle co-sedimentation assay (CSA), large (typically 100–200 nm) unilamellar, single bilayer liposomes with defined lipid composition are prepared, filled with raffinose solution, and incubated with a protein of interest, again generated recombinantly or by *in vitro* translation (Julkowska *et al.*, 2013; Marković and Jaillais, 2022; Scholz *et al.*, 2022). Subsequent high-speed centrifugation separates the sedimented vesicles and associated proteins into a pellet, and the unbound proteins remain in the supernatant. Both fractions are then subjected to SDS–PAGE, and the protein of interest is detected either by protein-staining dyes such as Coomassie brilliant blue or by western blotting (Julkowska *et al.*, 2013; Herianto *et al.*, 2022). CSA outcome can provide information about the lipid-binding specificity of the studied protein and affinities and stoichiometries of mutual interaction (Table 1). One of the limitations of this method is the preparation of liposomes, as they are sensitive towards lipid phase separation and should be composed of unsaturated lipids with identical acyl chain composition to achieve ideal lipid mixing. Moreover, CSA is not particularly suitable for studying large proteins or protein complexes, as they spontaneously sediment to the pellet during the centrifugation step (Julkowska *et al.*, 2013).

An alternative liposome-based method, which alleviates the problem of large protein sedimentation, is the co-flotation assay (CFA), also called the liposome binding assay. Similarly to CSA, CFA is based on the incubation of unilamellar liposomes with the protein of interest, but the separation of liposome-bound proteins from unbound proteins is achieved by centrifuge-induced flotation of the liposome-bound proteins on a sucrose cushion through the stepwise sucrose gradient (typically 0–30%). The top fraction harboring the bound proteins and the lower fractions are then subjected to SDS–PAGE and analyzed in the same manner as in CSA (van Galen *et al.*, 2012; Tronchere and Boal, 2017; Linster *et al.*, 2020; Liu *et al*., 2020). It should be noted that compared with the liposome sedimentation assay, CFA is more laborious due to time-demanding preparation and handling of the gradient fractions, constraining it to a limited number of experimental variations.

One common inherent caveat of previously described techniques is their inability to analyze the lipid–proteins interaction for integral membrane proteins or proteins with lipidic membrane anchors. The classical liposome-based assays also fail to explore possible membrane fusion events facilitated by the studied protein and do not provide information on the dynamics and kinetics of lipid–protein interactions (Senju *et al.*, 2021). To overcome these limits, a liposome turbidity assay (LTA, also called light scattering assay), which measures the change in absorbance due to protein-induced liposome fusion or aggregation, was introduced (Roston *et al.*, 2011). The results are then calculated as absolute absorbance values with subtraction of the absorbance measured prior to protein addition (Roston *et al.*, 2011; Ufer *et al.*, 2017; Qian *et al.*, 2022). The change in the diameter and clustering of liposomes can then be analyzed by TEM (Roston *et al.*, 2011). Such a need for subsequent analyses also illustrates one limit of the LTA method, which is inherently indirect, and it may be challenging to infer the underlying molecular details from the turbidimetry changes alone.

The majority of previously mentioned biochemical methods are low-throughput approaches. However, as myriads of possible lipid and protein interactions may occur in any living system, high-throughput platforms are necessary to study their interactions. Analogously, variations of the classical techniques that offer better quantitative outcomes are needed (Herianto *et al.*, 2022). In the following part, we present a succinct overview of recently established methods with possible implementation in plant cell biology (see also Table 1 for additional information).

One high-throughput technique that enables the analysis of a large number of proteins with selected lipids is based on proteome microarrays, an emerging technique that allows a systematic and comprehensive proteome-wide characterization of biomolecule responses in a single experiment. Proteome microarrays consist of a support surface (usually a chemically modified glass slide), to which an array of thousands of proteins, often a whole proteome, has been covalently immobilized in a high-density format. The tested liposomes (either biotinylated or fluorescently labeled) are incubated with the proteome array, and a laser scanner records the interactions between the liposomes and candidate proteins immobilized on the microarrays. Indeed, this approach was for the first time successfully used to map the global protein–lipid interactome of >5000 proteins and five phosphoinositide members in yeast (Zhu *et al.*, 2001). While proteome chip technology is in principle also available for Arabidopsis (Syu *et al.*, 2020), thus offering possible application of this technique for plant research, the complex chip manufacturing and the need for special readout hardware may limit its widespread use.

Another high-throughput technique based on the array principle is the liposome microarray-based assay (LiMA). This approach is essentially complementary to the protein microarray as it allows the quantitative analysis of multiple liposome preparations with selected peripheral membrane protein candidates (Saliba *et al.*, 2014). In recent LiMA applications, the array consists of tens of different giant unilamellar liposomes (up to 120), self-assembled in four separate microfluidic chambers fixed to the thin agarose layer. A protein with a fluorescent tag is loaded onto the device and allowed to associate with lipids, and the interaction is captured by an automated microscope. In principle, LiMA enables unparalleled high-throughput analysis of protein–lipid interaction in a biologically relevant environment, allowing the analyses of cooperative lipid binding, as demonstrated for 91 fungal and animal PH domains (Vonkova *et al.*, 2015). On the other hand, the disadvantage of LiMA (and also the proteome microarray method) is the lengthy preparation of the arrays, which also requires specific equipment and demands a certain level of expertise, and challenging data analysis (Herianto *et al.*, 2022).

An additional liposome-based method to be exploited in the plant field is proximity-based labeling of membrane-associated proteins (PLiMAP). This technique is a modification of the CSA approach and is based on activation of a synthesized photoactivable fluorescent lipid reporter (BODIPY diazirine PE, BDPE) by UV light, which cross-links BDPE with proteins bound to membranes and renders them fluorescent (Table 1). Typically, candidate proteins are incubated with liposomes with custom composition but containing a low amount (1%) of BDPE and, after cross-linking, it is subjected to SDS–PAGE. Gels are first imaged for fluorescence using green fluorescent protein (GFP) filters and then stained using colorimetry. The protein’s affinity for selected lipids is evaluated according to the strength of the fluorescence signal in the gel (Jose and Pucadyil, 2020; Jose *et al*., 2020). While PLiMAP suggests improved sensitivity over canonical CSA and CFA, it does not directly report the fraction of total protein bound to the membrane, which is estimated using classical liposome assays. Moreover, the BDPE cross-linking probe may also affect the protein–lipid interaction due to the presence of a diazirine group in the headgroup and a hydrophilic BOPIDY moiety in one acyl chain.

### Affinity purification with immobilized lipids

Using lipids covalently bound to chromatography matrices to identify novel lipid-binding proteins is one of the classical techniques established >20 years ago (Manifawa *et al.*, 2001). This technique typically involves the preparation of lipid-coated matrices (for some lipids, these are commercially available), and the lipid-containing beads are then incubated with the proteins of interest (i.e. cell lysate). After washing the unbound fraction, proteins that specifically bind to the immobilized lipid can be eluted and collected for further analysis. This approach thus allows the identification of lipid-binding proteins from complex biological samples, as it is compatible with MS. Notably, the application of this approach led to the identification of multiple PA-binding proteins in Arabidopsis (Testerink *et al.*, 2004). However, the lipids in this assay are not present in a physiologically accurate state, which may lead to either lack of binding for some candidate proteins that require a specific membrane environment or non-specific binding due to the artificial presentation of lipids on beads.

## Beyond the naked eye: unraveling lipid–protein interactions with biophysical approaches

Several classical biophysical techniques were modified to allow the study of lipid–protein interactions. Compared with biochemical methods, they differ in the principles of detection that are based on the changes in physical properties following lipid–protein binding such as resonance, wavelength, frequency, or temperature. Typically, lipids are present here in the form of a supported monolayer or as immobilized liposomes, often commercially available. These approaches are acclaimed for enabling real-time monitoring of lipid–protein interaction and, as label-free techniques, there is no need for labeling the lipid/protein with a fluorescent or other tag. Moreover, they also allow quantitative data (kinetics, binding affinity, enthalpy, stoichiometry, etc.) to be determined. By contrast, highly specialized and expensive instrumentation is necessary to operate these measurements. Due to the need for a highly technically demanding background and expertise of the operating staff for these methods, here we will present only a brief description pinpointing their main strengths and weaknesses, and provide recent references to the selected examples, that are already used or have a potential to enhance the research of lipid–protein interactions in plant biology (Fig. 1B; Table 1).

Surface plasmon resonance (SPR) is the golden standard of optical-based approaches. Lipids are immobilized as artificial membranes or liposomes on the gold-covered sensor chip surface where injected protein sample (typically at several different concentrations) is flowing across the surface employing a microfluidic system. Upon binding, changes in resonance properties of surface plasmons, that are equal to the mass concentration at the sensor chip surface, are recorded by the detector. A single experiment cycle is finished by regeneration of the sensor chip. SPR requires a small amount of sample (SPR sensitivity is at picomolar levels of proteins) and allows relatively fast quantification of association and dissociation kinetics, binding affinity, and thermodynamic data in real time. One could recognize the limitations of SPR as expensive instrumentation, requirements of high maintenance of the traditional microfluidics due to clogging, and non-specific surface binding of some analytes (Šakanovič *et al.*, 2019).

Bio-layer interferometry (BLI) is another optical-based technology suitable for describing lipid–protein binding. Lipids are present in the form of immobilized liposome that are prepared using sterically stabilized micelles (Wallner *et al.*, 2017). The BLI instrument shines white light onto the sensor tip surface and then analyzes its interference pattern reflected from two biosensor tip surfaces: a layer of immobilized molecules on the biosensor tip and an internal reference layer. Protein binding to the biosensor tip causes a shift in the interference pattern which directly correlates with the thickness of the layer on the surface of the biosensor (Wallner *et al.*, 2013). BLI can determine specificity, binding kinetics, and affinity of lipid–protein interaction in real time. In contrast to SPR, the BLI technique is established in an open shaking micro-well plate without any microfluidics, meaning there is no risk for clogging effects. Moreover, unbound molecules or sample buffer do not affect the interference pattern, allowing analysis of crude or complex samples. On the other hand, measurements are limited by diffusion and are less sensitive than SPR (Wallner *et al.*, 2013).

Quartz-crystal microbalance (QCM) is a high-resolution mass sensor in a low-throughput flowthrough system that detects the changes in frequency of a quartz crystal resonator. Lipids, in the form of immobilized liposomes or a supported lipid bilayer, are attached to the sensor chip surface, which is coated by SiO_2_ and sandwiched between a pair of gold electrodes (Nielsen and Otzen, 2019). Interaction between lipids and proteins results in the increase of mass at the sensor surface and leads to a detectable decrease in the oscillation frequency of the crystal. Binding kinetics are recorded in real time and the affinity rate constant is derived. Using lipid layers and in combination with dissipation monitoring, QCM is suitable for analysis of complex binding events such as conformational changes, fibrillation, and hierarchical clustering on the surface, which is difficult to interpret with conventional surface sensor techniques (Birchenough and Jowitt, 2021). Interactions of proteins from complex samples can also be measured as the optical properties of samples have no effect on the measurement. On the other hand, the signal of unspecific binding has to be subtracted using two channel measurements.

Isothermal titration calorimetry (ITC) is a biophysical method for measuring the binding and dissociation of the lipid–protein complex in a low- to medium-throughput manner. Usually, no immobilization of lipids (and proteins) is required, which ensures measurements of binding in their native states in solution. An ITC instrument consists of an identical sample and reference cell (filled with water or buffer) with high thermal conductivity and surrounded by an adiabatic jacket (Swamy *et al.*, 2019). One binding partner is placed in the sample cell, which is then titrated by adding the second binding partner through an injector syringe. ITC works by directly measuring the heat that is either released or absorbed during the binding event, resulting in accurate determination of binding constants, stoichiometry, entropy, and enthalpy in a single experiment. The disadvantages of this method include requirements for a high amount of pure binding partners, soluble in the same buffer system.

## Seeing is believing: genetically encoded lipid sensors as the key ingredients for the microscopic assessment of lipid–protein interactions

**Fig. 2.**
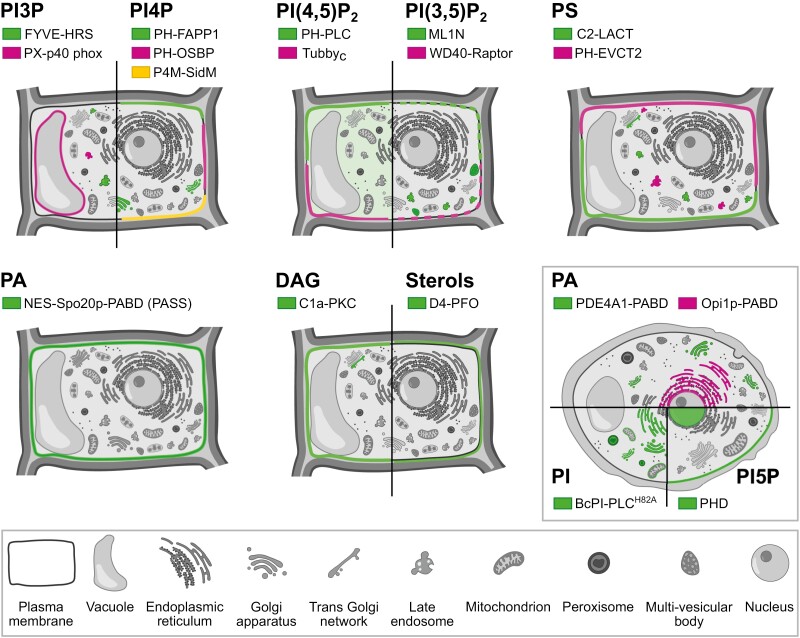
Graphical summary illustrating the typical subcellular localization pattern of lipid-specific genetically encoded biosensors employed for live cell microscopy. Please note that detailed localization patterns for individual sensors may slightly differ depending on the tandem organization (i.e. 1×, 2×, or 3×). The boxed inset depicts the localization of biosensors that still need to be employed in plant cells. See text for more details.

While the biochemical and biophysical approaches highlighted above are instrumental in detecting and quantifying lipid–protein interactions, they often provide only a static snapshot of such interactions outside the cellular context. It is therefore necessary to bolster the biochemical experiments with complementary methodology that captures the dynamic aspect of cellular membranes and the interactions within. Further, it is paramount to verify protein–lipid interactions in a native, cellular context to correctly decipher their biological functions. Consequently, microscopic tools that can visualize specific lipids and manipulate lipid production with high specificity are essential for elucidating biologically relevant protein–lipid interactions. Here, employing live-cell microscopic techniques allows us to visualize the localization, relative abundance, and dynamics of individual lipid classes together with the protein of interest in a spatio-temporal manner (Noack *et al.*, 2019). In the following sections, we briefly update recent advances and tools available for microscopic localization and *in vivo* manipulation of selected anionic membrane lipids (see Figs 1C and 2, and Table 1).

### Phosphoinositides

Phosphoinositides are a membrane lipid class characterized by an anionic inositol head group phosphorylated by specific lipid kinases at various positions. Five out of seven possible phosphoinositides are present in plants based on their biochemical detection and corresponding biosynthetic enzymes (Heilmann and Heilmann, 2022). Currently, >20 phosphoinositide biosensors are available (Hammond *et al.*, 2022), and several have already been introduced in plants (Simon *et al.*, 2014). Below is a selected list of state-of-the-art phosphoinositide sensors verified in plant cells and several new promising candidates (Fig. 2).

The most frequently used markers for phosphatidylinositol 3-phosphate (PI3P) are based on highly specific binding of FYVE finger or PX domains from mammalian proteins localizing to early endosomes. Interestingly, the two markers do not show the exact co-localization as 2×FYVE-HRS primarily located at late endosomes/pre-vacuolar compartment (PVC), while 1×PX-p40phox also showed additional localization to the tonoplast (Vermeer *et al.*, 2006; Simon *et al.*, 2014; Ito *et al.*, 2021a). Whether this discrepancy reflects different avidities of the two probes or whether 1×PX-p40phox binds to other 3-phosphorylated phosphoinositides at the tonoplast remains to be established.

To visualize PI(3,5)P_2_, a tandem repeat of the cytosolic phosphoinositide-interacting domain ML1N is most frequently used and decorates lysosomes and vacuoles in yeast and animal cells (Li *et al*., 2013). Similarly, in Arabidopsis, 2×ML1N localization corresponds to the accumulation of PI(3,5)P_2_ in the late endosomes/multivesicular bodies (MVBs), although the sensor has spatio-temporal limitations solely to the roots (Hirano *et al*., 2017). Surprisingly, 2×ML1N and another PI(3,5)P_2_ marker, called WD40-Raptor, decorated the restricted area of the plasma membrane of the root hair shank, suggesting possible cell type-specific localization (Hirano *et al*., 2018). However, the validity of 2×ML1N as a PI(3,5)P_2_-specific probe was challenged by Hammond *et al.* (2015), and the exact PI(3,5)P_2_ localization site thus remains open.

PI4P is among the most abundant phosphoinositides in most eukaryotic cells, including plants, constituting ~0.2–0.5% of cellular phospholipids (Balla, 2013; Bahammou *et al.*, 2024). In yeast and animal cells, PI4P localizes predominantly to the Golgi apparatus and, to a lesser extent, to the plasma membrane (PM) and late endosomes (Balla, 2013). Strikingly, already the first reports describing PI4P localization in plants using the 1×PH-FAPP1 sensor reported robust PM localization of PI4P in addition to the Golgi and *trans*-Golgi network (TGN) (Vermeer *et al*., 2009). This was further corroborated by observation with higher avidity variants containing two (2×PH-FAPP1) or three (3×PH-FAPP1) PH domains (Simon *et al.*, 2016; Ito *et al.*, 2021a). Consistently, both the other probes 1×PH-OSBP and P4M-SidM confirmed the predominant PM localization of PI4P in plants (Simon *et al.*, 2016).

The PH domain of human PLCδ1 is the canonical PI(4,5)P_2_ biosensor used across eukaryotic kingdoms. The lower avidity single variant (1×PH-PLC) localizes weakly to the PM and strongly to the cytoplasm in most studied cell types (van Leeuwen *et al.*, 2007; Simon *et al.*, 2014), in contrast to the high avidity tandem reporter (2×PH-PLC), which almost exclusively decorates the PM (Simon *et al.*, 2014, 2016). The C-terminal domain of the TUBBY protein, another available PI(4,5)P_2_ biosensor, is localized predominantly to the PM (Simon *et al.*, 2014; Caillaud, 2019) while also staining nuclei, pointing to the possible nuclear role of this phosphoinositide (Gerth *et al.*, 2017).

While the subcellular distribution of most phosphoinositides is well described in plant cells, two missing pieces of the puzzle remain. First, PI5P is a very minor component of phosphorylated phosphoinositides detected biochemically upon stress treatment (Meijer *et al.*, 2003). In animal cells, a triple version of the PHD domain from the ING2 protein localizes predominantly to the nucleus (Gozani *et al.*, 2003), and the modified tandem variant [glutathione *S*-transferase(GST)–2×PHD] weakly decorated the PM (Pendaries *et al.*, 2006). Interestingly, the PHD domain of Arabidopsis trithorax protein (ATX1) seems to bind PI5P comparatively, if not better, to the ING2-PHD domain and localizes to both the nucleus and PM, indicating its promise for the plant PI5P sensor (Alvarez-Venegas *et al.* 2006). Second, very little is known about the subcellular localization of PI, an abundant lipid (Colin and Jaillais, 2020) and the precursor of all phosphoinositides. Recently, the first genetically encoded sensor for PI detection in animal cells was described, consisting of engineered catalytically inactive bacterial PI-specific phospholipase C (PI-PLC) (Pemberton *et al.*, 2020; Zewe *et al.*, 2020). Surprisingly, the PI-PLC sensor indicated the presence of PI at the cytosolic leaflet of the endoplasmic reticulum (ER), Golgi, peroxisomes, and mitochondria, but extremely low PI levels at the PM (Fig. 2B). Since lipidomic analyses of biochemically purified plant PM preparations show high levels of PI (5–10% of glycerolipids, Furt *et al.*, 2011; Serrano *et al.*, 2022), it will be interesting to employ a PI-PLC sensor also in plant cells.

### Phosphatidylserine

The subcellular localization of PS is predominantly evaluated by either the stereospecific C2 domain of bovine Lactadherin (C2-LACT) or the PH domain of human EVECTIN2 (PH-EVCT2, typically as a 2×PH tandem arrangement) because they were extensively validated as calcium-independent PS reporters in animal, yeast, and plant cells. Both biosensors localize to the PM and secretory vesicles, the TGN, and along the endocytic pathway in various tissues (Platre *et al*., 2018; Kubátová *et al.*, 2019).

### Phosphatidic acid

Compared with bulky headgroup-containing phosphoinositides and PS, for which multiple specific domain folds were described, microscopic visualization of the simplest phospholipid PA is challenging. Moreover, PA is not only an important signaling phospholipid, but also a crucial intermediate in biosynthetic pathways for many other phospholipids, and resides in multiple cellular pools. Currently, variants of the PA-binding domain (PABD) from the yeast SNARE Spo20p are the most commonly used PA biosensor in both plant tissues and animal/yeast cells (Potocký *et al.*, 2014; Kassas *et al.*, 2017). The enhanced variants of 1× or 2×Spo20p-PABD including the nuclear export signal (NES) sequence and nicknamed PASS strictly localize to the inner leaflet of the PM (Platre *et al*., 2018; Pejchar *et al.*, 2020; Kalachova *et al.*, 2022). Since Spo20p-derived markers show affinity solely for PA in the PM, the necessity for biosensors for other PA pools arises. In yeast and animal cells, the PABD domain from a yeast transcriptional repressor, Opi1p, mainly binds ER or nuclear PA (Hofbauer *et al.*, 2018). Furthermore, PABD from mammalian PDE4A1 shows specific affinity for the PA pool in the Golgi (Kassas *et al.*, 2017; Żelasko and Czogalla, 2022). Selectivity of different PA markers for distinct subcellular PA pools then depends on PA acyl chain length and saturation, but also on global membrane packing defects, net membrane charge, or the presence of sterols in the membrane (Kassas *et al.*, 2017). All those specificity factors are also present in plant endomembranes, making these biosensors promising subjects for further exploitation in plants.

To improve the quantitative aspect of PA imaging, Li *et al*. (2019) designed a ratiometric plasma membrane PA probe, which is based on PABD from Arabidopsis NADPH oxidase RbohD inserted between cyan fluorescent protein (CFP) and Venus fluorescent proteins and anchored to the PM. Upon PA binding, conformational changes of this sensor (termed PAleon) lead to a measurable fluorescence resonance energy transfer (FRET) signal. The experiments with PAleon also indicated that changes in cellular pH may lead to protonation-induced changes in PA charge and thus affect PA functions (i.e. protein binding).

### Diacylglycerol

Compared with non-plant models, where diacylglycerol (DAG) is an essential second messenger regulating protein kinase C activity, DAG in plants was long considered to function merely as a by-product of PI(4,5)P_2_ degradation. However, recent advances in the biology of plant diacylglycerol kinases (DGKs) have sparked new interest in the subcellular localization of DAG in plant cells. To this day, a single type of DAG biosensor, based on Cys1 domains from mammalian protein kinases PKC and PDK1, has been described. In animal cells, the Cys1 biosensor revealed DAG on the Golgi, ER, and nuclear envelope, while in plants, the majority of the Cys1 signal resides on the PM and TGN (Vermeer *et al.*, 2017; Scholz *et al.*, 2022).

### Sterols

Sterols are important components of all eukaryotic membranes, constituting ~10% of whole-cell lipids and up to 30% of PM lipids. The plant sterol family encompass a large diversity of molecular species in both free and conjugated forms (Bahammou *et al.*, 2024). Only relatively recently, a cholesterol biosensor based on the point-mutated D4 domain of theta-toxin perfringolysin O (PFO) from the bacterium *Clostridium perfringens* and termed D4H was developed, showing the presence of intracellular sterols on the PM and endomembranes (Maekawa and Fairn, 2015). These observations were corroborated by a novel sensor based on the fungal protein maistero-2, which seems to be more sensitive than D4H. Notably, the maistero-2 sensor was also shown to bind a large set of 3-hydroxylated sterols *in vitro*, including major plant sterol species (Yamaji-Hasegawa *et al.*, 2022). Recently, an apoplastic variant of the D4 sensor (SP-sfGFP-D4L) was utilized in plant cells for the first time, revealing a pattern of nanoscale-sized puncta on the PM surface (Ukawa *et al.*, 2022).

## Outside masters: exogenous lipid and lipid inhibitor treatments for *in vivo* validation of protein–lipid interactions

In higher plants, most genes coding for lipid biosynthetic enzymes and lipid kinases or phosphatases are present in multiple paralogs (except for the PS biosynthetic enzyme PSS1; see below). Coupled with the often-seen compensatory effects, this makes genetic approaches for studying individual roles of distinct lipid classes (or their combinations) in membrane recruitment of peripheral proteins particularly challenging. An alternative strategy to test this is to use lipid-specific inhibitors (Table 2). A typical application is to analyze the recruitment of a protein of interest to the target membranes upon inhibitor treatment by quantifying the membrane/cytoplasm ratio (Iswanto *et al.*, 2020; Vogel *et al.*, 2022; Wattelet-Boyer *et al.*, 2022).

**Table 2.** Inhibitors frequently used to manipulate plant lipids *in vivo*

Lipid	Enzyme	Inhibitor	Dosage/treatment time	References^*a*^
PI4P	PI-4 kinase	Phenylarsine oxide (PAO)	30–60 μM/15–30 min	Barbosa *et al.* (2016); Platre *et al*. (2018)
		Wortmannin	33 μM/45–90 min	Vogel *et al.* (2022)
PI3P	PI-3 kinase	Wortmannin	1 μM/30–60 min	Simon *et al.* (2016)
		LY294002	50–100 μM/30–60 min	Tan *et al.* (2020)
PA	DAG kinase	R59022	12.5–25 μM/30–60 min	Kalachova *et al.* (2022); Platre *et al*. (2018)
	PLD	*n*-Butanol	0.2–0.5%/10–60 min	Hunter *et al.* (2019); Tan *et al.* (2020)
	LPAAT	CI-976	5–10 μM/>24 h	Wattelet-Boyer *et al.* (2022)
DAG	PLC	U73122	1–5 μM/90 min	Ito *et al.* (2021b)
Sterols	CPI1	Fenpropimorph	50 μg ml^–1^/10–24 h	Stanislas *et al.* (2015); Synek *et al.* (2021)
	HMG-CoA reductase	Lovastatin	1 μM/>24 h	Kulich *et al.* (2024); Stanislas *et al.* (2015)
Sphingolipids	SPT	Myriocin	0.1 μM/12–24 h	Iswanto *et al.* (2020)

^*a*^ Only selected examples employing the lipid inhibitors to study protein recruitment to membranes are listed.

The effect of lipid-dependent protein recruitment to cellular membranes can be further exploited by external addition of phospholipids. Here, using more soluble lysophospholipids, namely lyso-PA and lyso-PS, has been proven to be particularly useful. Furthermore, lysophospholipid add-back experiments can also be performed together with the application of inhibitors (Platre and Jaillais, 2021). Notably, this setup also allows testing the lipid redundancy—protein dissociation from the membrane after PI4P inhibition by phenylarsine oxide (PAO) is reverted by the addition of lyso-PA (Synek *et al.*, 2021).

The pharmacological approaches described above are well established and used in plant cell research. Below, we will mention several recently developed pharmacological tools that can provide novel information on lipid–protein interactions *in vivo* with much greater detail. One such approach is the application of bifunctional lipid-derived affinity-based probes (AfBPs), which can be utilized to map protein–lipid interactions in living cells in detail (Shanbhag *et al.*, 2023). AfBP probes provide two features: a UV light-photoactivatable covalent linkage to neighboring proteins and a click chemistry tag to enable the detection of cross-linked proteins and their enrichment for subsequent identification by MS (Ge *et al.*, 2022). Indeed, the application of fatty acid AfBPs coupled with MS/MS proteomics led to the description of proteome-wide identification of lipid-binding proteins in mammalian cells (Niphakis *et al.*, 2015).

Another example of a photoconversion-based method are photo-caged lipid probes, where a native lipid is rendered inactive by a chemical modification, which introduces a photoremovable group (e.g. BODIPY) that is cleaved upon light exposure. The caging group can also dictate the localization of the lipid probe in subcellular structures. Upon photoactivation, the caging group is released, and triggers an acute concentration increase of now active lipids in distinct cellular membranes. A major advantage is that the concentration changes can be induced rapidly and at precise locations within subcellular membranes (Farley *et al.*, 2021). A possible drawback of this method is the side effects caused by the photoreaction in the compartment being investigated, which can lead to unintended consequences if high probe concentrations are required. Similarly, the presence of high levels of caged lipid probes might affect the biochemical properties of living biological membranes (Jiménez-López and Nadler, 2023).

## Inside job: employment of genetically encoded lipid-modulating enzymes

Pharmacological treatment with lipid inhibitors and exogenous lipids or lipid analogues is an effective approach, but it also has inherent constrains. In addition to the possible non-specific action of the inhibitors and the often prohibitive price of lipid analogs or their commercial unavailability, one of the main drawbacks is often the insufficient penetration to deeper plant tissues, limiting the pharmacological tools to epidermal cells. To overcome these limitations and to establish a more fine-tuned way to modulate the cellular lipid levels, genetic systems based on tunable activation of lipid-depleting or lipid-generating enzymes were established.

The simplest iteration of this approach is the chronic overexpression of endogenous lipid enzymes. For example, the overexpression of PI(4,5)P_2_-generating PIP kinases PIPK10/11 or PA-generating PLDδ3 in pollen tubes was employed to study the role of PI(4,5)P_2_ or PA, respectively, in mechanisms of pollen tube growth (Ischebeck *et al*., 2011; Pejchar *et al.*, 2020).

Another possibility is the constitutive expression of engineered lipid-modifying enzymes targeted into distinct endomembrane compartments via specific localization motifs. This approach was used to manipulate levels of PI4P at the PM, utilizing the catalytic domain of the yeast PI4P phosphatase Sac1p [which specifically dephosphorylates PI4P without impacting PS or PI(4,5)P_2_], and PM targeting through a myristoylation and palmitoylation (MAP) sequence. Indeed, the co-expression of MAP-Sac1p together with a PI4P marker perturbed the PM localization of the sensor and the signal was translocated to the endosomes, validating the impact of Sac1p on natural pools of PI4P (Simon *et al.*, 2016; Gronnier *et al.*, 2017).

An analogous approach was implemented to establish the inducible depletion of PI(4,5)P_2_ in plants (iDePP), employing a synthetic inducible system designed to specifically dephosphorylate PI(4,5)P2 at the PM. It consists of the inducible expression of the isolated phosphatase domain from *Drosophila melanogaster* inositol polyphosphate 5-phosphatase OCRL, fused with a fluorescent tag and directed to the PM by a MAP sequence. Indeed, expression of iDePP led to the complete dissociation of the PI(4,5)P_2_ biosensor 2×PH-PLC 3 h after induction. Remarkably, iDePP had no impact on the localization and levels of other anionic phospholipids, which makes it a specialized tool for PI(4,5)P_2_ depletion. iDePP was successfully used to prove the PI(4,5)P_2_-dependent membrane recruitment of the AP-2 endocytic adaptor complex (Doumane *et al.*, 2021).

A unique genetic system in Arabidopsis phospholipid biology are the knockout mutants in *PHOSPHATIDYLSERINE SYNTHASE1* (*PSS1*), a gene required for the final step of PS biosynthesis in plants (Yamaoka *et al.*, 2011). Importantly, knockout mutants *pss1-3* or *pss1-4* produce no PS, with only minor changes in other phospholipids. Notably, the existence of the *pss1-3* mutant was instrumental for the validation of PS probes C2-LACT and 2×PH-EVCT2, the development of the lysophospholipid add-back assay, and the discovery of PS-dependent nanodomain clustering of the small GTPase ROP and its molecular targets (Platre and Jaillais, 2021; Smokvarska *et al.*, 2023).

## Conclusion

After decades of neglect, cellular membranes and their lipid components are no longer considered simple hydrophobic barriers, and membrane lipids are now being recognized for their diverse interactions with integral and peripheral membrane proteins, which have deep effects on their biological functions. To understand these complex phenomena and unravel the molecular details behind the generation and function of biological membranes, an array of multifaceted experimental approaches for identifying and elucidating protein–lipid interactions needs to be employed. In this review, we have summarized selected biochemical, biophysical, and cell biological approaches to detect protein–lipid interactions and investigate the underlying mechanistic details in plants. We emphasize the need to concurrently use multiple *in vitro* and *in vivo* techniques to obtain a global and coherent picture of the protein–lipid interactions in a biological context. Simultaneously, we call for adopting newly emerged techniques that can further revolutionize plant membrane research. Specifically, there is an urgent need to employ methods enabling proteome-wide identification of lipid-binding proteins in plant cells and to characterize the individual interactions quantitatively. At the same time, we need to introduce novel lipid biosensors and state-of-the-art lipid manipulation tools. The employment of optogenetic tools, which will enable acute and precisely localized changes in levels of specific membrane lipids, is paramount. Further developing sensitive, high-throughput, and cell type- and membrane-specific techniques for mapping plant protein–lipid interactomes on the molecular scale will boost our understanding of plant cell membranes.

## Data Availability

This review contains no new experimental data.
